# Synthesis, optical, and structural properties of bisphenol-bridged aromatic cyclic phosphazenes *Dedicated to supervisor Prof. Dr. Adem Kılıç on his retirement.


**DOI:** 10.3906/kim-1907-73

**Published:** 2020-02-11

**Authors:** Bünyemin ÇOŞUT, Burcu TOPALOĞLU AKSOY, Süreyya Oğuz TÜMAY, Ahmet ŞENOCAK, Serkan YEŞİLOT

**Affiliations:** 1 Department of Chemistry, Faculty of Science, Gebze Technical University, Gebze, Kocaeli Turkey

**Keywords:** Cyclotriphosphazene, cyclic voltammetry, fluorescence, DFT, bisphenol A

## Abstract

Phenoxy- and naphthoxy-substituted bisphenol-bridged cyclic phosphazenes were synthesized in 2 steps and their thermal, photophysical, and electrochemical properties were investigated. The structures of the cyclic phosphazene compounds were determined by ESI-MS mass spectrometry and ^1^ H, ^13^ C, and ^31^ P NMR spectroscopies. The photophysical studies of phenoxy- and naphthoxy-substituted bridged cyclophosphazenes were investigated by means of absorption and fluorescence spectroscopies in different solvents. Thermal and electrochemical properties of the target compounds were also studied. Furthermore, the excimer emissions through intramolecular interactions in solution and in solid state were investigated by fluorescence spectroscopy and the theoretical calculations were performed in detail using DFT.

## 1. Introduction

In recent years, there has been great interest in the development of new fluorescent materials for many applications, such as light-emitting devices [1], biomedical studies, [2], molecular sensors [3], and solar energy [4]. It is well known that several aromatic hydrocarbons (e.g., benzene, naphthalene, and anthracene) for preparing fluorescent materials are used as themselves or starting materials for new π-conjugated materials [5–7]. In general, they are prepared as small molecules [6,7], dimers [8], or oligomers/polymers [9,10], and for extending π-electron systems. There are many examples of their utility as fluorescent materials in the scientific literature [11]. One reason for further research on aromatic hydrocarbons is that they have high stability and quantum efficiency, leading to the development of fluorescence molecules. Hence, there remains great interest in the design and synthesis of new fluorescence materials using some aromatic hydrocarbons, such as benzene and naphthalene, which were selected herein as new materials.

Cyclic phosphazenes are an important inorganic group of compounds that can be easily substituted using nucleophilic compounds such as phenols, alcohols, and amines via halogen atoms, which are bound to phosphorus atoms [12–15]. They are also important precursors for specialty polymers [16,17]. Due to the chemical and thermal stability of the cyclic phosphazene skeleton, they have various applications, such as biomedical materials [18], flame retardants [19], liquid crystals [20], elastomers [21], or lubricants [22]. Furthermore, cyclophosphazenes have been placed in hybrid polymer electrolytes for lithium ion batteries [21–23]. Aminocyclotriphosphazenes and ferrocenylphosphazenes are 2 of the varying derivatives of phosphazenes. Aminocyclotriphosphazenes have shown great antimicrobial activity against bacteria and fungi [21] and ferrocenylphosphazenes have exhibited cytotoxic activity against cancer cells [14,21]. Therefore, new heterocyclic cyclophosphazene structural types have gained attention in both academic and industrial communities [24]. Additionally, cyclic phosphazenes are optically inert in the UV-Vis region; hence, photophysical properties can be adjusted by substituted groups on the phosphorus atoms [13,25–27]. In this manner, cyclic phosphazene- or polyphosphazene-based fluorescent compounds have been prepared for the development of luminescent materials [25,28–30]. Cyclic phosphazenes can vary according to reaction with mono-, bi-, and polydentate reagents. Reaction with difunctional ligands results in spiro-, dispiro-, trispiro-, ansa-, spiro ansa-, or intermolecular products [15,16,21,31].

The precursors N _3_ P _3_ Cl _6_ (trimer) and N _4_ P _4_ Cl _8_ (tetramer) are the most commonly known cyclic phosphazenes and are commercially available. Moreover, owing to optical inertness in the UV-Vis region, they are excellent platforms for the preparation of fluorescent materials for numerous applications, such as fluorescent sensors [32], light-emitting devices [29], electrochromic materials [25], and coordination polymers [33].

In this study, the molecular design, synthesis, and characterization of phenoxy- and naphthoxy-substituted bridged cyclic phosphazenes (Figure 1) are presented, which were obtained by nucleophilic substitution reactions of bisphenol A and cyclic phosphazenes 1–4 at ~80% yields, and their thermal, optical, electrochemical, and structural properties were investigated.

**Figure:**
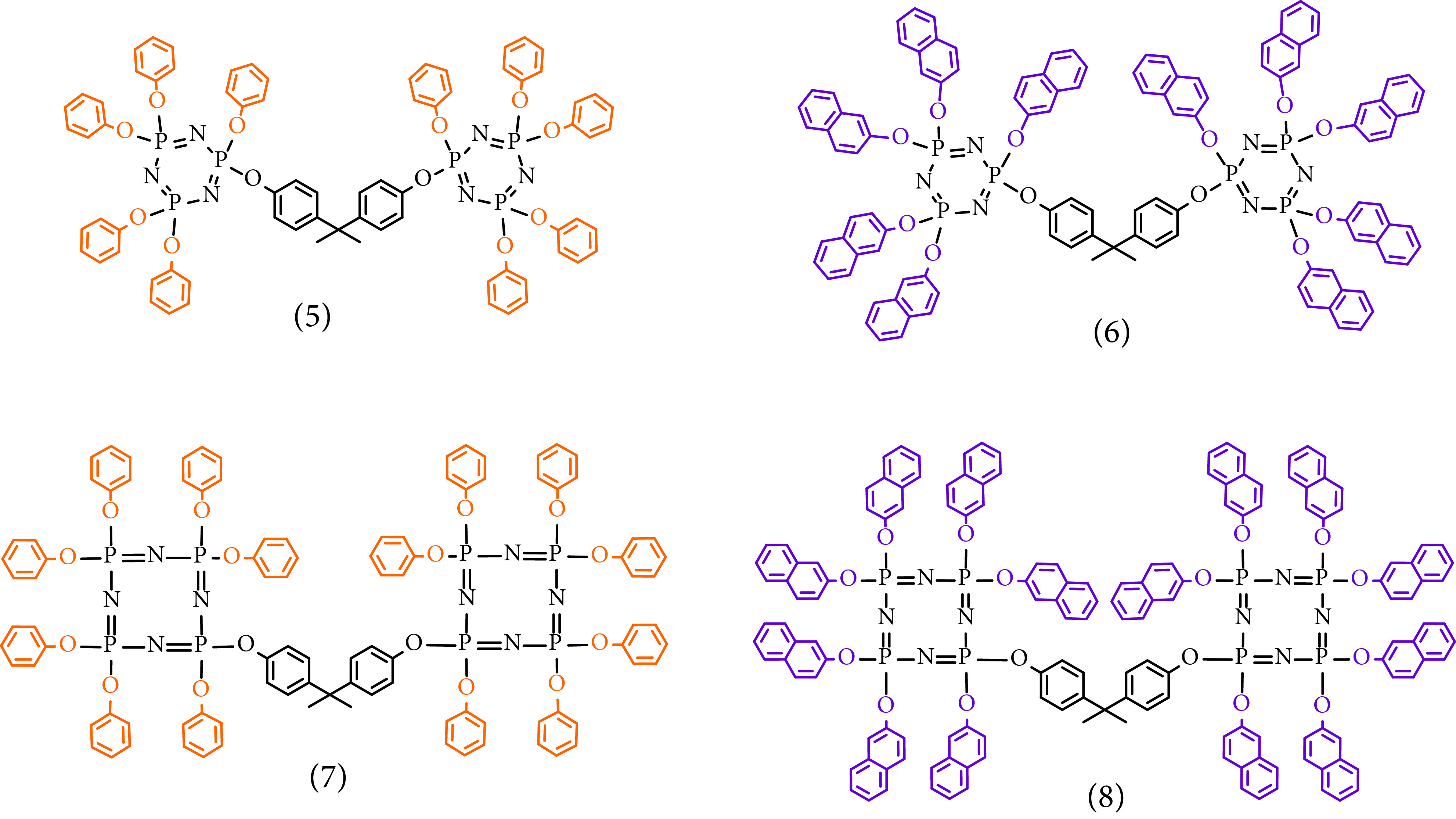
Molecular structures of 5–8.

## 2. Materials and methods

All reagents were used without further purification and purchased from Sigma-Aldrich (St. Louis, MO, USA), and all solvents were obtained from Merck (Darmstadt, Germany). Reactions were monitored by TLC using Merck TLC silica gel 60 F254. Silica gel column chromatography was used over Merck silica gel 60 (particle size: 0.040–0.063 mm, 230–400 mesh ASTM). ^1^ H, ^13^ C, and ^31^ P NMR spectra were obtained for all of the compounds in CDCl3 on a Varian INOVA 500 MHz spectrometer (West Sussex, UK) using TMS as an internal reference for the ^1^ H and ^13^ C NMR measurements. Thermal properties of the compounds were recorded with a Mettler Toledo TGA/SDTA 851 thermogravimetric analysis (TGA) instrument (Columbus, OHý, USA) at a heating rate of 10 °C/min under nitrogen. Electronic absorption spectra in the UV-Vis region were measured with a Shimadzu 2101 UV-Vis spectrophotometer (Tokyo, Japan). Fluorescence excitation and emission spectra were obtained on a Varian Eclipse spectrofluorometer (Melbourne, Australia), using 1-cm path-length cuvettes at room temperature. The fluorescence lifetimes were obtained using a Horiba Jobin-Yvon-SPEX Fluorolog 3-2iHR instrument with a Fluoro Hub-B single-photon counting controller (Kyoto, Japan) at an excitation wavelength of 310 nm for compounds 5–8. Signal acquisition was performed using a TCSPC module.

### 2.1. Parameters for fluorescence quantum yields

To determine the fluorescence quantum yields (Φ _F_ ) for the target compounds (5–8), the comparative calculation method was used [34].

(1)ϕF=ϕFstdF.AStdn2FStd.A.nStd2

In this equation, F and FStd represent the total area below the fluorescence emission spectra of compounds 5–8 and the standard. A and AStd are the absorbance of compounds 5–8 and the standard at the excitation wavelength. To calculate the fluorescence quantum yield, refractive indices (n) of the solvents were used due to the dissolution of compounds 5–8 and the standard in different solvents. As one of the most widely used standards in the literature, quinine sulfate (Φ _F_ = 0.54), which was dissolved in 0.1 M H _2_ SO _4_ , was used to calculate the fluorescence quantum yields of compounds 5–8 [35]. The final concentrations of compounds 5–8 were fixed at the same value as the quinine sulfate at the excitation wavelength. Fluorescence lifetimes of compounds 5–8 were directly measured and calculated with monoexponential calculations.

### 2.2. Synthesis

Compounds 1–4 were synthesized and purified according to literature procedures [5,36–39], respectively.

#### 2.2.1. Synthesis of compound 5

In a 100-mL 3-necked reaction flask, bisphenol A (0.06 g, 0.3 mmol) and Cs _2_ CO _3_ (0.3 g, 0.6 mmol) were added to compound 1 (0.855 g, 0.6 mmol), dissolved with 25 mL of dry THF. The mixture was stirred under reflux for 72 h and the progress of the reaction was monitored by TLC. The reaction mixture was separated from its salts by filtration through a G4 filter. The solvent of the filtrate (THF) was removed by means of a rotary evaporator. The resulting oily product was purified with column chromatography using THF and n-hexane (1:1) as the mobile phase. Yield: 0.34 g (80%). IR (ATR, room temp.): 1172 cm ^−1^ (P=N); 943 cm ^−1^ (P-O). MALDI TOF (m/z) calc. 1426.30, found: 1427.55 [M ^+^ ]. ^1^ H NMR (CDCl _3_ ) d = 7.85–6.53 (m, 58H, ArCH), 3.77 (br, 6H, CH _3_ ) ; ^13^ C NMR (CDCl _3_ ) d = 152.05 (ArC), 147.34 (ArC), 146.85 (ArC), 135.90 (ArC), 130.01 (ArCH), 124.07 (ArC), 118.4 (ArCH), 78.5 (ArCH), 68.6 (ArCH _3_ ) , ^31^ P NMR (toluene-d8) d: 9.83 (br, s, 3P).

#### 2.2.2. Synthesis of compound 6

In a 100-mL 3-necked reaction flask, bisphenol A (0.06 g, 0.3 mmol) and Cs _2_ CO _3_ (0.3 g, 0.6 mmol) were added to compound 2 (1.15 g, 0.6 mmol), dissolved with 25 mL of dry THF. The mixture was stirred under reflux for 72 h and the progress of the reaction was monitored by TLC. The reaction mixture was separated from its salts by filtration through a G4 filter. The solvent of the filtrate (THF) was removed by means of a rotary evaporator. The resulting oily product was purified with column chromatography using THF and n-hexane (1:1) as the mobile phase. Yield: 0.47 g (81%). IR (ATR, room temp.): 1190 cm ^−1^ (P=N); 946 cm ^−1^ (P-O). MALDI TOF (m/z) calc. 1926.46, found: 1928.55 [M ^+^ ]. ^1^ H NMR (CDCl _3_ ) d = 7.74–6.88 (m, 58H, ArCH), 3.80 (br, 6H, CH _3_ ) ; ^13^ C NMR (CDCl _3_ ) d = 151.85 (ArC), 148.54 (ArC), 147.05 (ArC), 136.15 (ArC), 129.6 (ArCH), 125.57 (ArC), 117.9 (ArCH), 77.5 (ArCH), 68.3 (ArCH _3_ ) , ^31^ P NMR (toluene-d8) d = 10.63 [m, 4P, >P(OPh) _2_ , B _2_ ], 10.02 [m,2P >P(OPh)(OPhO),A] ; 2 JP,P = 88.86 Hz.

#### 2.2.3. Synthesis of compound 7

In a 100-mL 3-necked reaction flask, bisphenol A (0.06 g, 0.3 mmol) and Cs _2_ CO _3_ (0.3 g, 0.6 mmol) were added to compound 3 (1.13 g, 0.6 mmol), dissolved with 25 mL of dry THF. The mixture was stirred under reflux for 72 h and the progress of the reaction was monitored by TLC. The reaction mixture was separated from its salts by filtration through a G4 filter. The solvent of the filtrate (THF) was removed by means of a rotary evaporator. The resulting oily product was purified with column chromatography using THF and n-hexane (1:1) as the mobile phase. Yield: 0.5 g (80%). IR (ATR, room temp.): 1187 cm ^−1^ (P=N); 945 cm ^−1^ (P-O). MALDI TOF (m/z) calc. 1888.39, found: 1890.82 [M ^+^ ]. ^31^ P NMR (toluene-d8) d: 11.28 (br, s, 4P). ^1^ H NMR (CDCl _3_ ) d: 7.29–6.88 (m, 78H, ArCH), 5.07 (br s, 6H, CH _3_ ) . ^13^ C NMR (CDCl _3_ ) d: 151.63 (ArC), 149.51 (ArCH), 146.64 (ArC), 136.07 (ArC), 129.43 (ArCH), 127.72 (ArCH), 125.81 (ArCH), 124.61 (ArCH), 121.27 (ArCH), 120.56 (ArCH), 68.21 (ArCH _3_ ) .

#### 2.2.4. Synthesis of compound 8

In a 100-mL 3-necked reaction flask, bisphenol A (0.06 g, 0.3 mmol) and Cs _2_ CO _3_ (0.3 g, 0.6 mmol) were added to compound 4 (1.55 g, 0.6 mmol), dissolved with 25 mL of dry THF. The mixture was stirred under reflux for 72 h and the progress of the reaction was monitored by TLC. The reaction mixture was separated from its salts by filtration through a G4 filter. The solvent of the filtrate (THF) was removed by means of a rotary evaporator. The resulting oily product was purified with column chromatography using THF and n-hexane (1:1) as the mobile phase. Yield: 0.65 g (80%). IR (ATR, room temp.): 1165 cm ^−1^ (P=N); 950 cm ^−1^ (P-O). MALDI TOF (m/z) calc. 2588.61, found: 2590.92 [M ^+^ ]. ^1^ H NMR (CDCl _3_ ) d: 7.71–6.65 (m, 86H, ArCH), 5.08 (br s, 6H, CH _3_ ) . ^13^ C NMR (CDCl _3_ ) d: 151.82 (ArC), 149.27 (ArCH), 146.7 (ArC), 136.1 (ArC), 134.06 (ArC), 131.01 (ArC), 129.44 (ArC), 127.85 (ArCH), 126.52 (ArCH), 125.81 (ArCH), 125.33 (ArCH), 120.40 (ArCH), 117.74 (ArCH), 68.26 (ArCH _3_ ) .

## 3. Results and discussion

### 3.1. Synthesis, characterization, and thermal analysis

**Figure 2:**
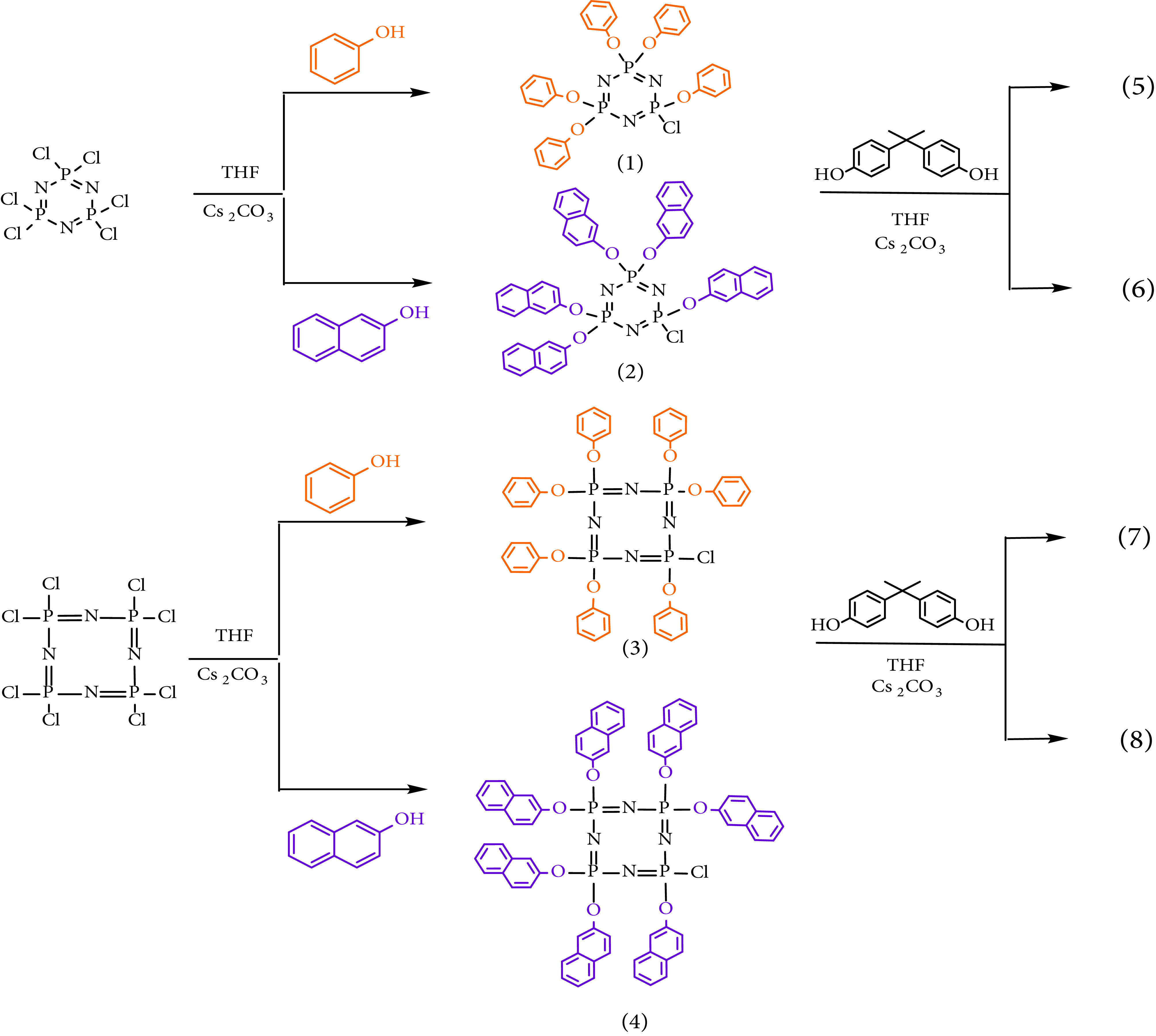
Synthetic route to compounds 1–8.

The synthetic pathways to compounds 1–8 presented in this work are shown in Figure 2. Compound 1 was synthesized by the reaction of phenol with hexachlorocyclotriphosphazene (trimer) in the presence of Cs _2_ CO _3_ in THF. Compound 2 was obtained by the reaction of 2-naphthol with trimer in the presence of Cs _2_ CO _3_ as the base in THF. For the synthesis of compounds 4 and 5, octachlorocyclotetraphosphazene (tetramer) was reacted with phenol and 2-naphthol, respectively. For the synthesis of bisphenol-bridged cyclophosphazene compounds, nucleophilic displacement reactions between the bisphenol and phenol and 2-naphthol decorated trimer and tetramer were performed under an argon atmosphere. The obtained phenol and 2-naphthol decorated cyclic phosphazene compounds were reacted with bisphenol at a 2:1 ratio in the presence of cesium carbonate in THF. The products were purified by preparative TLC on silica gel using hexane and THF (1:1) as the mobile phase.

The synthesized compounds were characterized by ^1^ H, ^13^ C, and ^31^ P NMR, mass and infrared spectrometry, and thermal analysis. All of the results were consistent with the predicted structures. The results are summarized in the Section 2 and the ^31^ P NMR and infrared spectra, thermal analysis, and mass results are given in the Supplemental information. In particular, the newly synthesized compounds were subjected to ^31^ P NMR spectroscopy analysis in order to confirm the targeted structures. For instance, the obtained ^31^ P NMR spectra (proton-decoupled) of compounds 5 and 7 showed A3 and A4 spin systems with the same chemical environment, respectively (Figures S1 and S2), which confirmed the suggested structures. It should be noted that the phenoxy units of the bisphenol, which were introduced into the bridgehead, had the same chemical environment as the other phenoxy-substituted phosphorus atoms. ^31^ P NMR spectra of compounds 6 and 8 were somewhat more complex, due to having different chemical environments than the phosphorus atoms. The ^31^ P NMR spectrum of compound 6 showed a typical AB2 spin system and gave rise to very close compound 8, and the calculated chemical shifts and coupling constants were at 10.63 ppm for >P(OPh), 10.02 ppm for >P(OPh)(OPhO), and 88.86 Hz (2 JP,P ) , respectively (Figures S3 and S4). As can be seen in Figure S4, the ^31^ P NMR spectrum of compound 8 was more complex and might be an AB2 C spin system; however, it was not possible to assign its coupling constants. In addition, the mass spectra of compounds 5–8 were consistent with the predicted structures (Figures S5–S8). According to the obtained FTIR spectra of the compounds, the P=N and P-O stretches were shown at 1172 cm ^−1^ and 943 cm ^−1^ for compound 5, 1190 cm ^−1^ and 946 cm ^−1^ for compound 6, 1187 cm ^−1^ and 945 cm ^−1^ for compound 7, and 1165 cm ^−1^ and 950 cm ^−1^ for compound 8, respectively (Figure S9).

TGA methods were used to investigate the thermal stability of all of the cyclophosphazene compounds (Figure S10). In the TGA analysis measurements of the compounds under nitrogen (N2) gas with heating at 10 °C/min up to 700 °C, the temperatures at which 5% decomposition for compounds 5–8 occurred were 465, 475, 460, and 477 °C, respectively.

### 3.2. Optical and electrochemical properties

**Figure 3:**
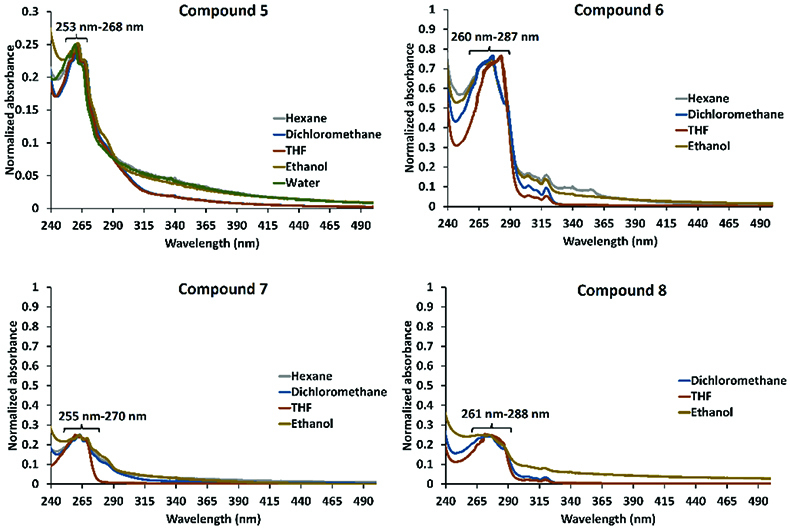
Normalized UV-Vis absorption spectra of compounds 5, 6, 7, and 8 in different solvents.

Photophysical properties of the phenoxy- and naphthoxy-substituted cyclic trimeric and tetrameric phosphazene compounds (5 and 6, and 7 and 8) were evaluated with UV-Vis absorption and fluorescence emission experiments. UV-Vis absorptions of the phenoxy-substituted cyclic trimeric and tetrameric phosphazene compounds (5 and 7) and naphthoxy-substituted cyclic trimeric and tetrameric phosphazene compounds (6 and 8) were measured in different solvents. As can be seen in Figure 3, compounds 5 and 7 demonstrated absorption maxima at 263 nm with an absorption band between ≈ 250 and 70 nm, whereas compounds 6 and 8 demonstrated absorption maxima at 272 nm with an absorption band between ≈ 260 and 288 nm. The observed absorption behaviors of compounds 5–8 can be attributed to the π-π* transitions of the phenoxy and naphthoxy groups, which appended on the cyclic trimeric and tetrameric phosphazenes [32,40–42]. This means that compounds 5–8 demonstrated similar absorption behaviors with phenoxy and naphthoxy, and this is a well-known phenomenon for phosphazene compounds, which are photophysically inert in the UV-Vis region [29,30]. In addition, these results showed that the phenoxy and naphthoxy groups on the cyclic trimeric and tetrameric phosphazenes had no effective ground state interaction.

**Figure 4:**
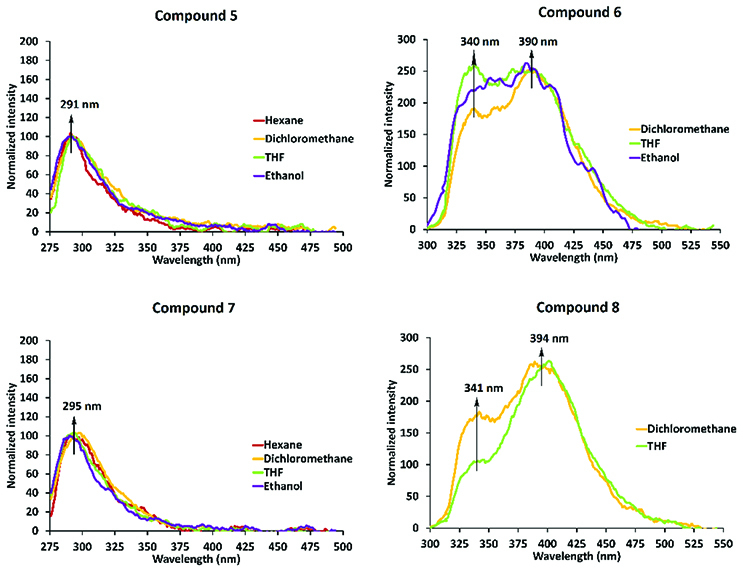
Normalized fluorescence emission spectra of compounds 5, 6, 7, and 8 in different solvents (λexc. : 260 nm for 5 and 7, and λexc. : 290 nm for 6 and 8).

After evaluation of the UV-Vis absorptions of compounds 5–8, their fluorescence emission behaviors were examined under the same conditions as in the absorption studies. Fluorescence emission spectra of the target compounds are given in Figure 4, in which compounds 5 and 7 were excited at 260 nm and compounds 6 and 8 were excited at 290 nm. According to Figure 4, normalized fluorescence spectra of 5 ×10−5 M for compounds 5 and 7 demonstrated classic monomer phenolic emission at ~290 nm [43], whereas under the same conditions, compounds 6 and 8 demonstrated excimer emission, which arose from the π-π stacking interaction between the naphthol units on the cyclic trimeric and tetrameric phosphazenes at ~390 nm. These red-shifted emission wavelengths of 6 and 8 increased up to 54 nm when compared to monomer naphthol emissions [44].

This bathochromic shift of compounds 6 and 8 was very similar to intermolecular excimer emission, and it is well known that intermolecular excimer formation is a concentration-dependent phenomenon and does not occur in dilute media [45]. In addition, the dissolution medium of compounds can affect monomer and excimer formation [46,47]. As can be seen in Figure 4, the monomeric phenol emissions of 5 ×10 ^-5^ M in compounds 5 and 7 and excimer emissions of 5 ×10 ^-5^ M in compounds 6 and 8 were not affected by the solvent system. According to the obtained results, the molar absorptivities of compounds 5–8 in different solvents were calculated and are given in Table 1.

**Table 1:** Molar absorptivity of compounds 5–8.

ε (L mol ^-1^ cm ^−1^ )	Hexane	Dichloromethane	THF	Ethanol	Water
5	3700	9600	8000	5000	2500
6	5300	54,000	55,000	10,000	1400
7	5400	11,000	9600	4200	1500
8	790	35,000	32,000	3400	1600

To evaluate the effect of concentration on the photophysical behaviors of compounds 5–8, the UV-Vis absorption and fluorescence emission spectra were examined at different concentrations in various solvents, such as hexane, dichloromethane (DCM), THF, ethanol, and water (Figures S11–S18). As can be seen in Figures S11–S18, compounds 5–8 were slightly soluble in hexane and water. In addition, the UV-Vis electronic absorption and fluorescence emission signals of compounds 5–8 decreased proportional to the concentration, and the monomeric phenol emissions of compounds 5 and 7 and excimer emissions of compounds 6 and 8 were consistent in diluted or concentrated media. These results showed that there was no intermolecular selfquenching, which was observed at high concentrations in compounds 5–8 [48]. Moreover, the excimer emission of compounds 6 and 8 was observed in a dilute concentration, which indicated that the intramolecular π-π stacking interaction between the naphthol units on the cyclic trimeric and tetrameric phosphazenes was the source of the excimer emission [32,46,47].

**Figure 5:**
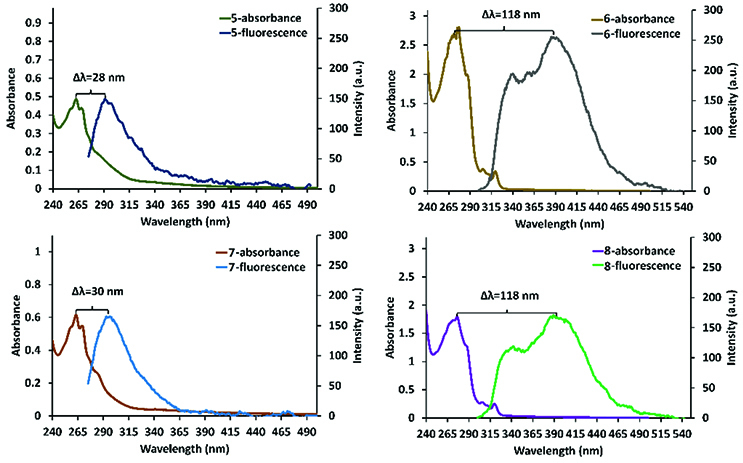
Stoke shifts of compounds 5, 6, 7, and 8 in DCM (λexc. : 260 nm for 5 and 7, and λexc. : 290 nm for 6 and 8).

**Figure 6:**
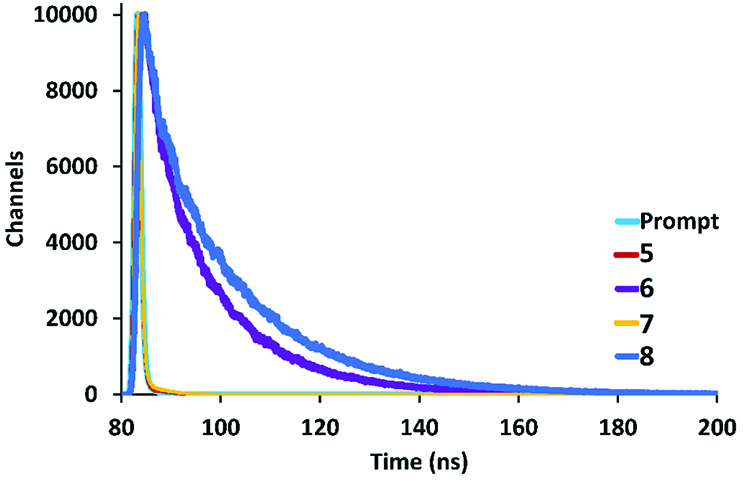
Fluorescence lifetime decays of compounds 5, 6, 7, and 8.

An important property of fluorescence dyes is a large Stokes shift, which reduces self-absorption, and this was calculated for compounds 5–8 in CH _2_ Cl _2_ and is given in Figure 5 [49]. According to Figure 5, the Stokes shifts of compounds 5 and 7 were calculated as 28 nm and 30 nm, respectively, whereas the Stokes shifts of compounds 6 and 8 were determined as 118 nm for both compounds. This large Stokes shift of compounds 6 and 8 can be attributed to intramolecular π-π stacking interactions between the naphthol units on the cyclic trimeric and tetrameric phosphazenes. The fluorescence lifetimes (τF ) of compounds 5–8 were calculated with monoexponential calculation as 0.131 ±0.007, 13.542 ±0.059, 0.147 ±0.008, and 17.143 ±0.045 ns, respectively (Figure 6). In addition, the fluorescence quantum yields (Φ _F_ ) of compounds 5–8 were calculated as 0.07, 0.29, 0.075, and 0.31, respectively, when quinine sulfate (Φ _F_ = 0.54) was used as the standard. The obtained results for the τF and Φ _F_ are given in Table 2.

**Table 2:** Fluorescence quantum yields (QF ) and lifetimes of compounds 5–8.

	5	6	7	8
QF (DCM)	0.070	0.280	0.075	0.310
τ (ns)	0.131 ±0.007	13.542 ±0.059	0.147 ±0.008	17.143 ±0.045

**Figure 7:**
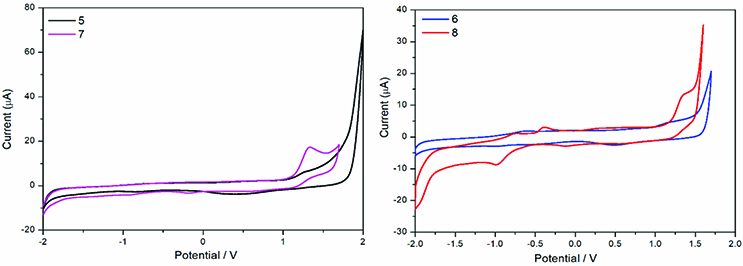
Cyclic voltammogram of compounds 5, 6, 7, and 8.

The cyclic voltammetry (CV) measurements were performed with the CH Instruments 440B electrochemical system (Bee Cave, TX, USA) (Figure 7). A glassy carbon electrode, Pt wire, and saturated calomel electrode served as the working, counter, and reference electrodes. Electrochemical grade tetrabutylammonium hexafluorophosphate (TBAPF6) in extra pure DCM was used as the supporting electrolyte in CV at a concentration of 0.10 mol dm−3 . High purity N2 was used for deoxygenation of the solution for 10 min prior to each run and maintained in a nitrogen blanket [50,51]. HOMO-LUMO levels and band gap values provide further information on the potential use of a new molecule for many applications, such as dye-sensitized and organic solar cells. HOMO-LUMO energy levels were determined by 3 different methods including UV-Vis absorption onset (λonset) and CV and DFT calculations. In the current study, the 3 methods used to estimate these values are summarized in Table 3.

**Table 3:** HOMO and LUMO energy level values determined from the experimental CV, theoretical calculations, and band gap energy values.

			5	6	7	8
Eox		1.24	1.13	1.23	1.32
EHOMO	CV	–5.64	–5.53	–5.63	–5.53
	DFT	–6.10	–5.55	–5.96	–5.54
ELUMO	UV/CV*	–1.44	–1.78	–1.50	–1.78
	DFT	–0.26	–1.13	–0.20	–1.07
Eg (cyclic)		3.12	2.99	3.05	2.99
Eg (optical)		4.20	3.75	4.13	3.75
Eg (DFT)		5.84	4.42	5.76	4.47

in the UV-Vis spectra of compounds 5–8 and was found as 4.20, 3.75, 4.13, and 3.75 eV, respectively, and then the LUMO could be estimated as –1.44, –1.78, –1.50, and –1.78 eV, respectively.

HOMO and LUMO energy levels of compounds 5–8 were calculated using the first oxidation and reduction values from CV. The corresponding HOMO energy levels of compounds 5–8 were calculated as –5.64, –5.53, –5.63, and –5.53 eV, respectively, using the equation EHOMO = – [(Eox – E1/2(ferrocene)) + 4.8]. The LUMO energy levels of these compounds were determined as –1.44, –1.78, –1.50, and –1.78 eV, respectively, using the UV-Vis spectra of these compounds. Band gap values of the compounds were determined as 3.12, 2.99, 3.05, and 2.99, respectively, from the electrochemical measurements. Moreover, band gaps were calculated from the UV-Vis absorption band onset (λonset) by the equation Eg (eV) = 1240/λonset (nm) and determined as 4.20, 3.75, 4.13, and 3.75 for compounds 5–8, respectively. HOMO and LUMO orbitals and energy gap levels were also calculated by performing single-point time-dependent DFT (TD-DFT) calculations using the B3LYP/6-31 G (d, p) levels (Table 4). HOMO-LUMO energies of compounds 5–8 are tabulated in Table 3 and the band gap values from the DFT calculation were determined as 5.84, 4.42, 5.76, and 4.47, respectively. These findings showed that these molecules were located at high energy levels and the large band gaps make these molecules potentially useful in electronics, light-emitting diodes, and solar cells.

**Table 4:** 

		
5	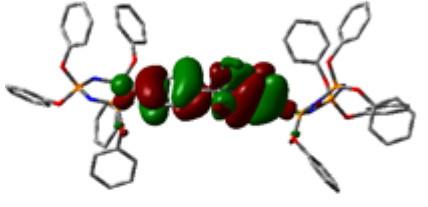	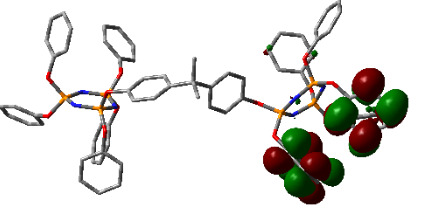
6	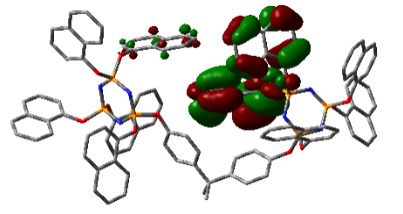	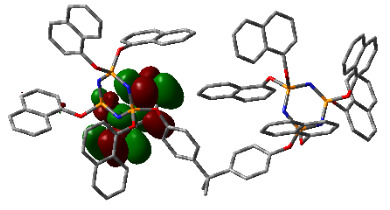
7	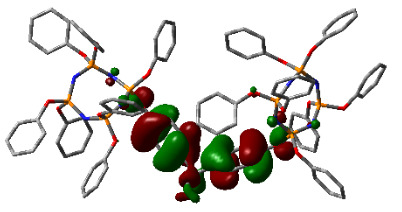	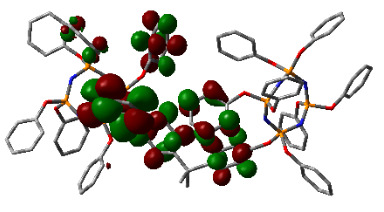
8	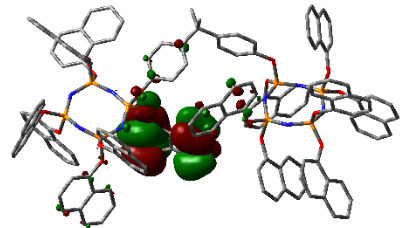	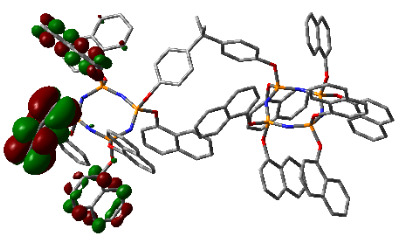

### 3.3. Computational results

**Figure 8:**
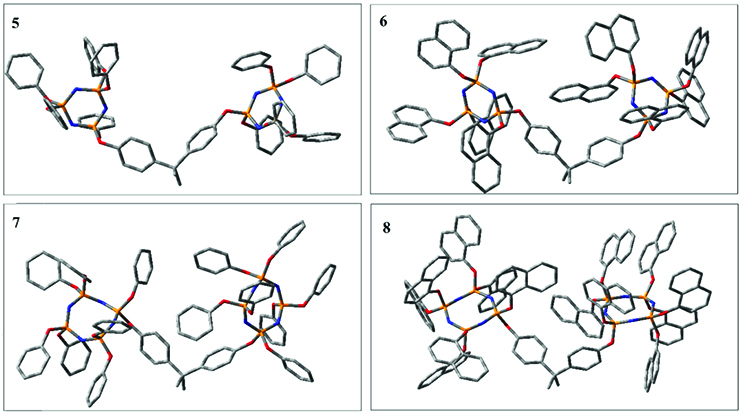
Optimized geometries of compounds 5, 6, 7, and 8 using B3LYP/6-31G (d, p) levels.

**Figure 9:**
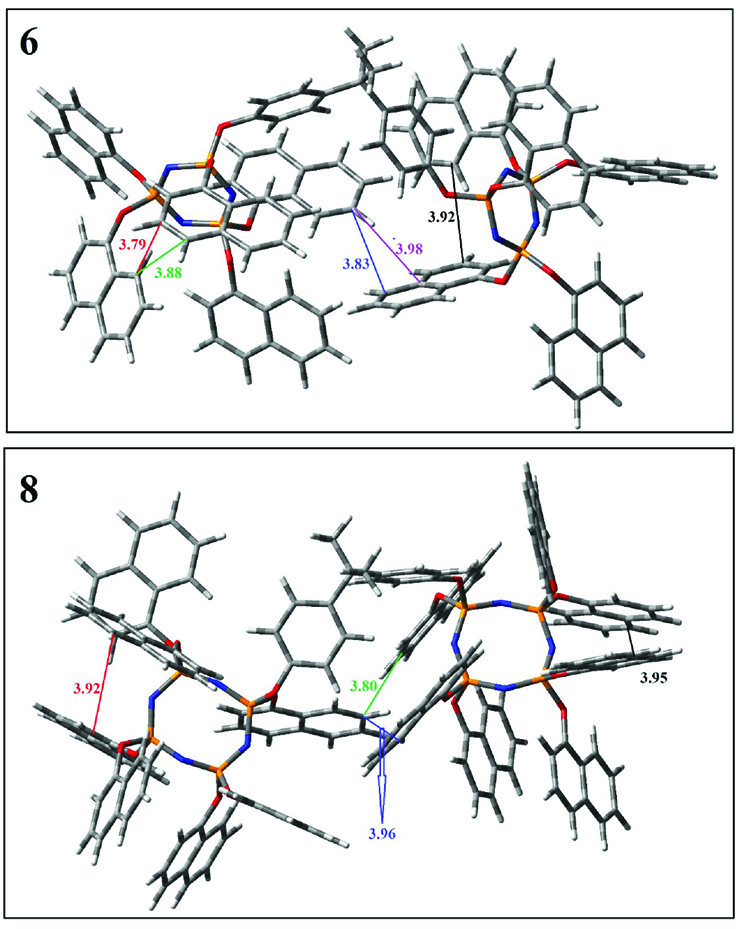
Selected intramolecular π -π interactions in the structures of compounds 6 and 8.

The Gaussian 09 package was used for all theoretical computations, which were performed via DFT to obtain more information about the optimized structures of the new compounds and their intramolecular interactions among the phenol and naphthol groups [52]. Geometry optimization studies were performed by B3LYP with a basis set of 6-31G (d, p) and the nearest values to the crystal structure could be obtained with this method [53]. The optimized geometries of compounds 5–8 are illustrated in Figure 8. Although phenoxysubstituted phosphazene molecules 5 and 7 did not exhibit any intramolecular interactions, naphthol-containing phosphazene compounds 6 and 8 exhibited intramolecular noncovalent interactions. The distances of the phenoxy and naphthoxy groups for compounds 6 and 8 are illustrated in Figure 9. The selected intramolecular naphthoxy groups were in the range of 3.79 to 3.98 Å, which is within the accepted range for π-π interaction of less than 4.0 Å [46]. According to the results of the DFT calculations, while excimer emissions occurred within the molecules in compounds 6 and 8, monomer emissions occurred in compounds 5 and 7, but they were not within the molecules, and the theoretical results were in perfect harmony with the fluorescence results.

## 4. Conclusions

To summarize, we presented the design, synthesis, and characterization of phenoxy- and naphthoxy-substituted bisphenol-bridged cyclic phosphazenes (5–8). Standard spectroscopic techniques, such as ESI-MS, ^1^ H, ^13^ C, and ^31^ P were used for the characterization of compounds 5–8. The thermal, optical, electrochemical, and structural properties of the target bridged cyclic phosphazenes (5–8) were investigated by TGA, UV-Vis, and fluorescence spectroscopies and CV and DFT calculations, respectively. According to the UV-Vis and fluorescence measurements, the naphthoxy-substituted bisphenol-bridged cyclic phosphazenes (6 and 8) showed excimer emissions while their phenoxy derivatives (5 and 7) demonstrated only monomer emissions at 290 nm, which was confirmed by theoretical calculations. In addition, the HOMO and LUMO orbitals and energy gap levels were calculated and confirmed by electrochemical measurements, UV-Vis absorption band onset, and single-point (TD-DFT) calculations. According to all of the investigations, compounds 5–8 are potential candidates for important industrial applications, such as light-emitting diodes, solar cells, and flame retardants.

Supplementary Materials

## Acknowledgments
