# Supplementary material for: Synthesis, optical, and structural properties of bisphenol-bridged aromatic cyclic phosphazenes
Source: Turk J Chem. 2020 Feb 11;44(1):48–63. doi: 10.3906/kim-1907-73 (PMC7751810; doi:10.3906/kim-1907-73)
Supplement: Supplementary file 1 — Supplementary Materials [file turkjchem-44-48-sup001.pdf]

Supplemental information

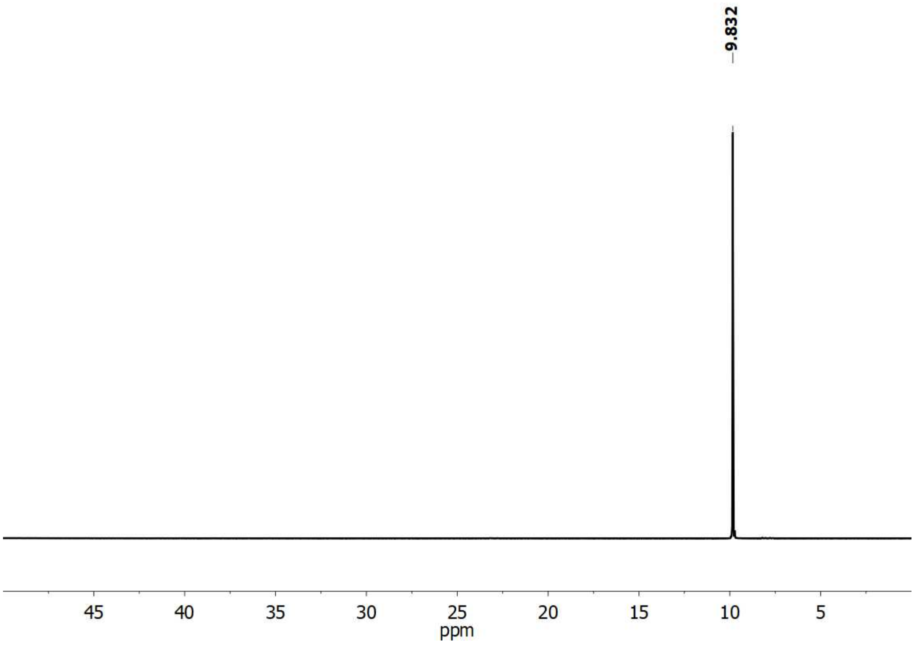

Figure S1. ^{31}P NMR spectrum of compound **5**.

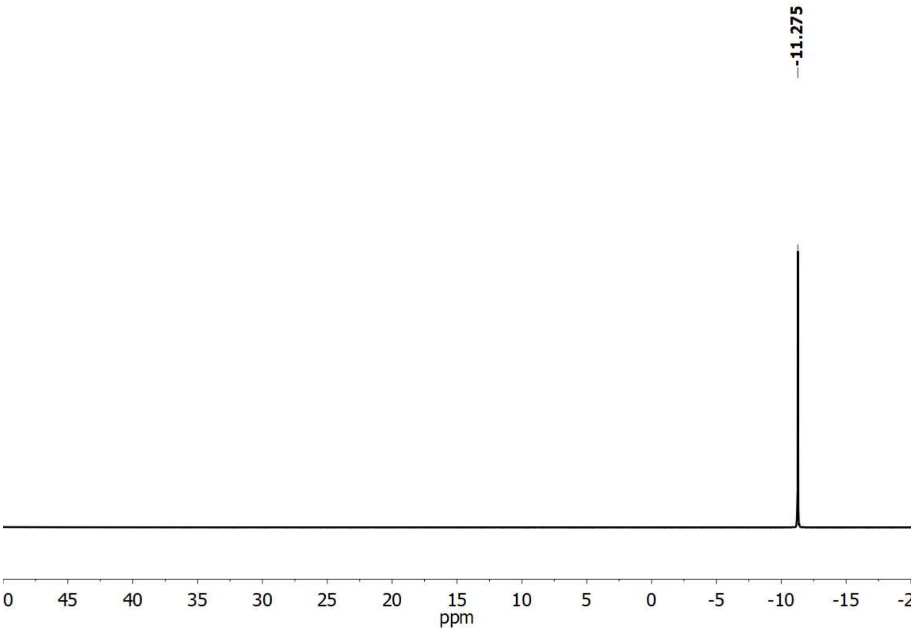

Figure S2. ^{31}P NMR spectrum of compound **7**.

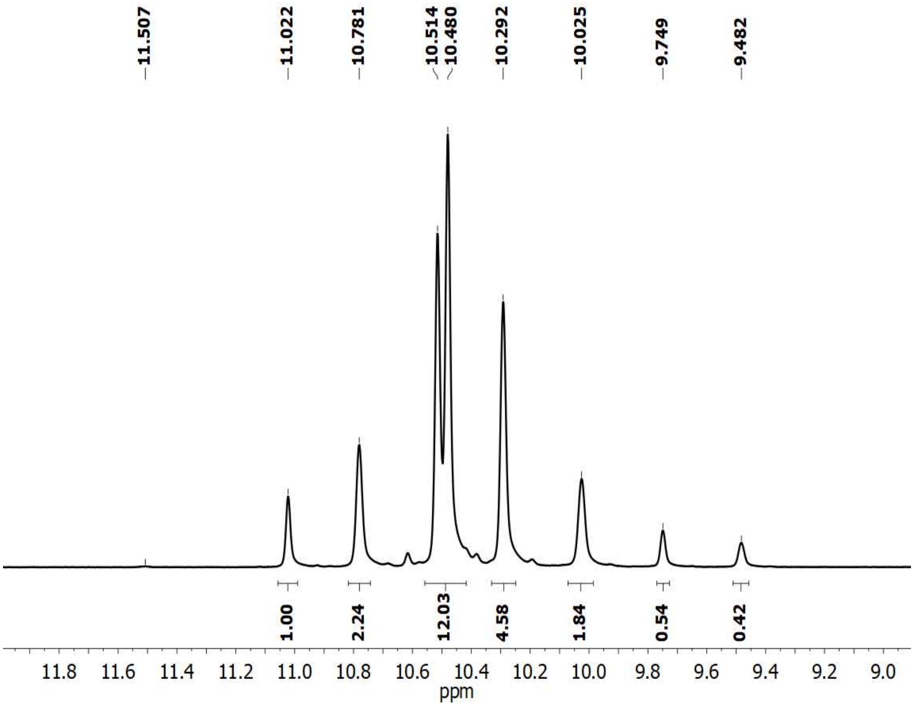

Figure S3. ^{31}P NMR spectrum of compound **6**.

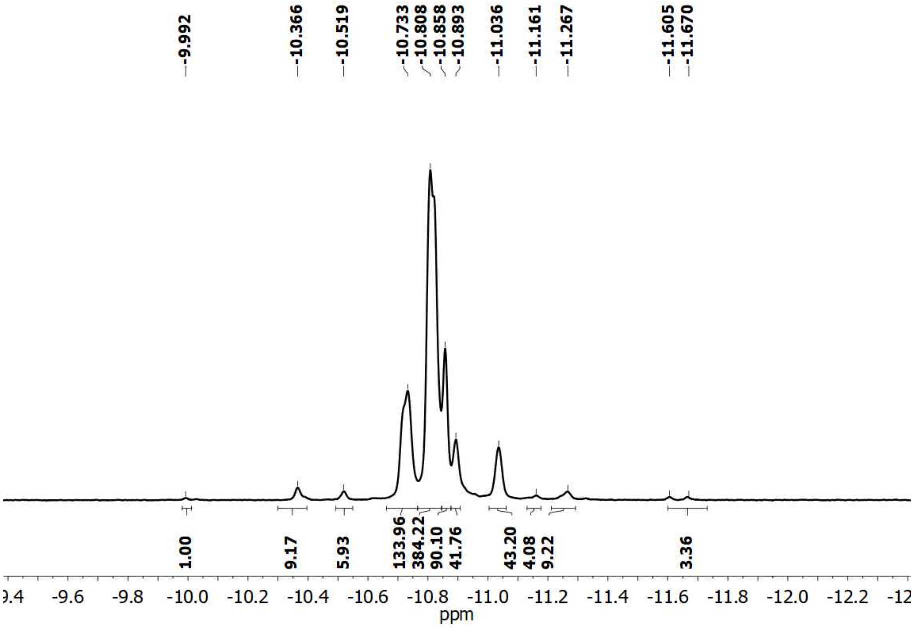

Figure S4. ^{31}P NMR spectrum of compound **8**.

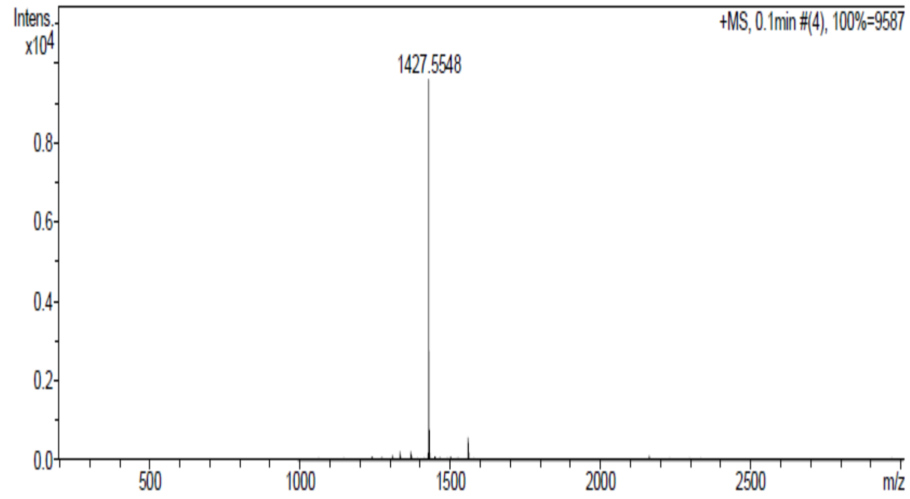

Figure S5. Mass spectrum of compound **5**.

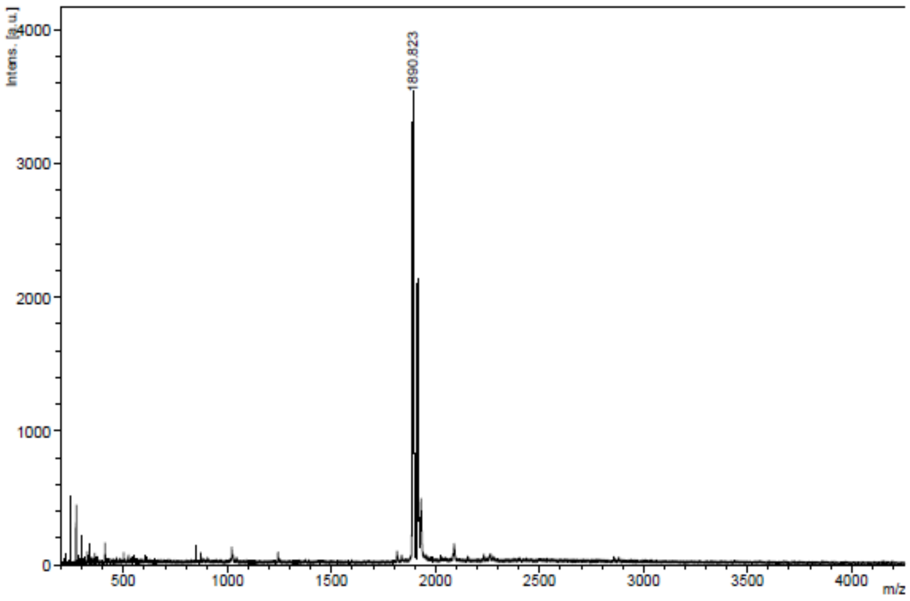

Figure S6. Mass spectrum of compound **7**.

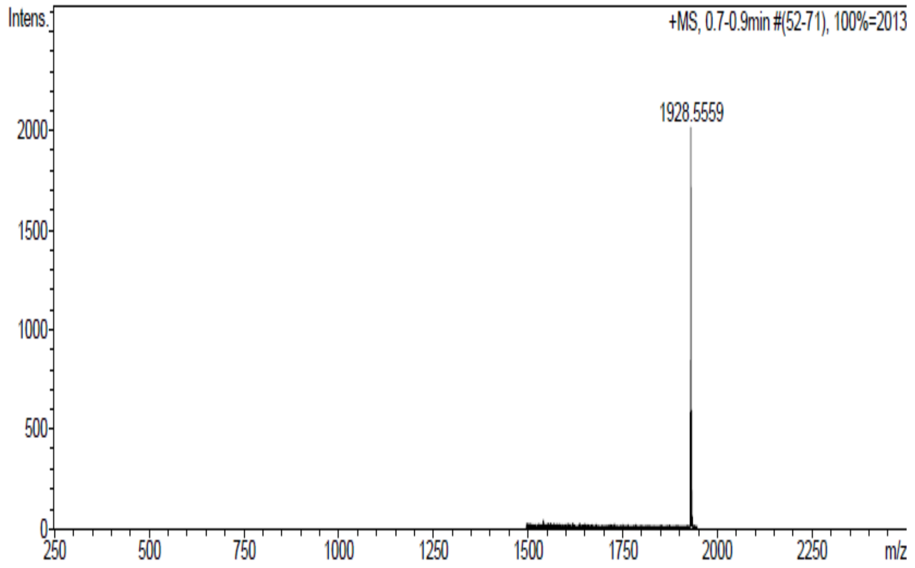

Figure S7. Mass spectrum of compound **6**.

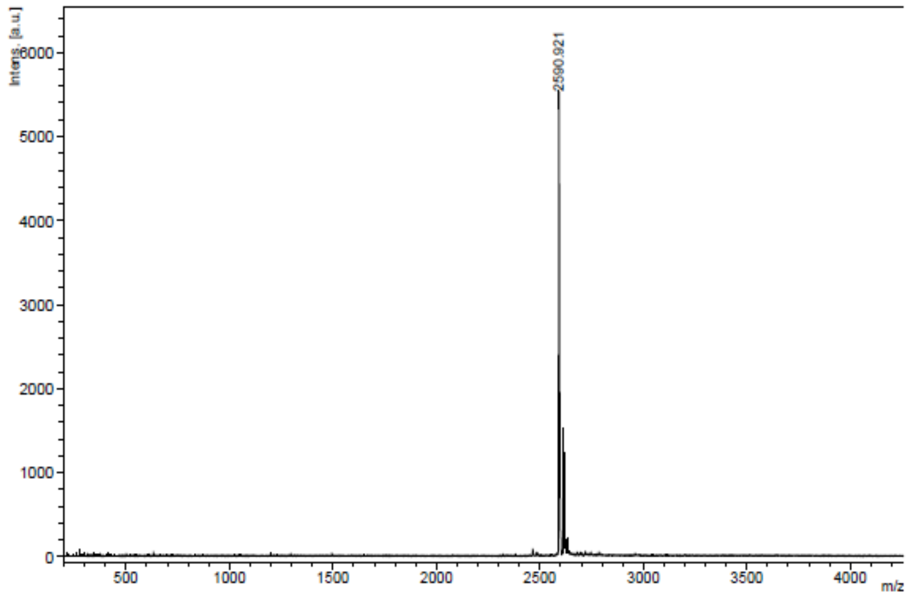

Figure S8. Mass spectrum of compound **8**.

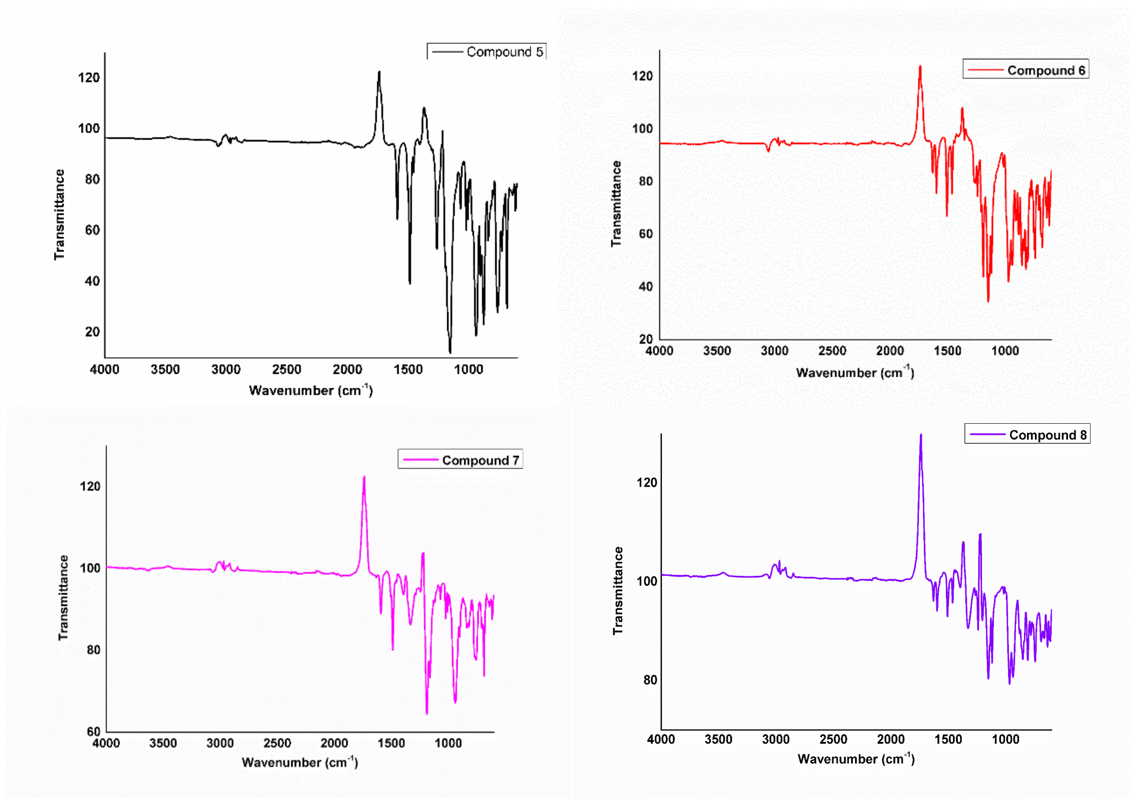

Figure S9. FTIR spectrum of compounds **5**, **6**, **7**, and **8**.

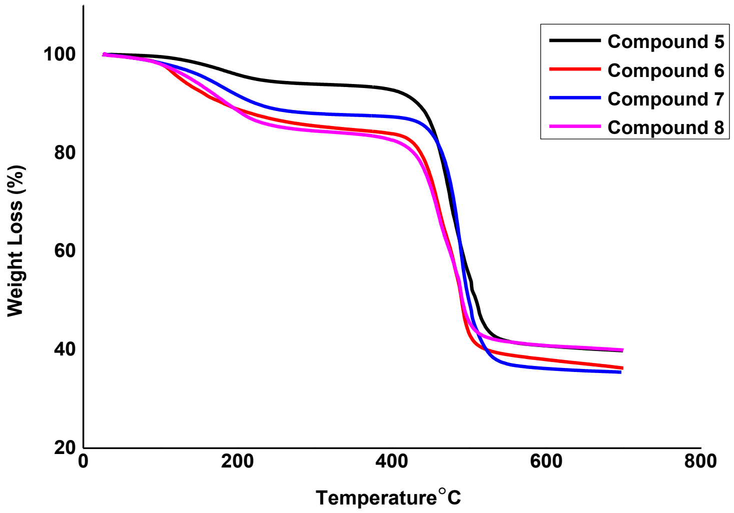

Figure S10. TGA thermograms of compounds **5**, **6**, **7**, and **8**.

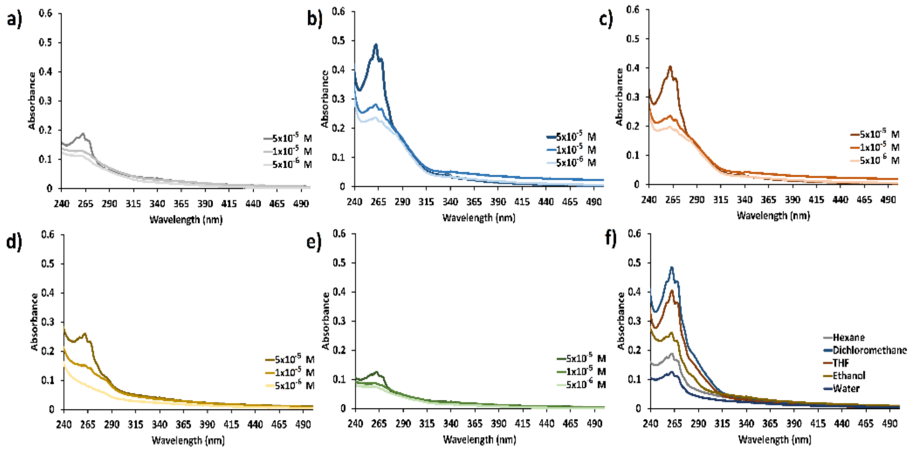

Figure S11. UV-Vis absorption spectra of compound **5** in (a) hexane, (b) DCM, (c) THF, (d) ethanol, and (e) water at various concentrations, and (f) overlap spectra of the solvents at 5 × 10^{-5} M.

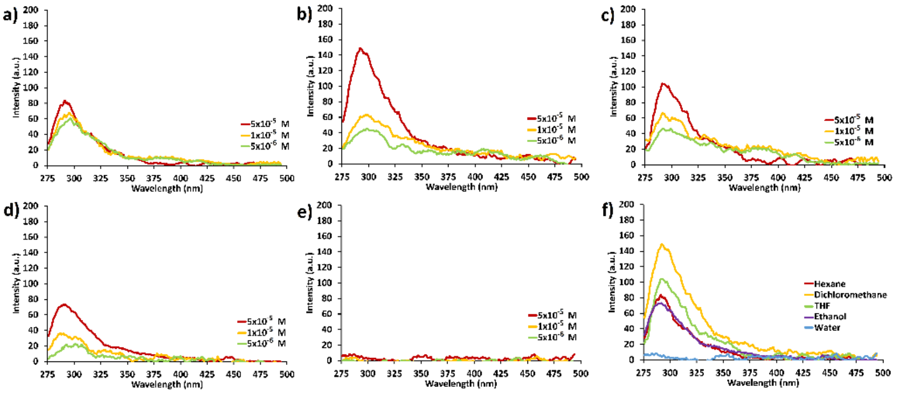

Figure S12. Fluorescence emission spectra of compound **5** in (a) hexane, (b) DCM, (c) THF, (d) ethanol, and (e) water at various concentrations, and (f) overlap spectra of the solvents at 5 × 10^{-5} M ($\lambda_{exc.}$: 260 nm).

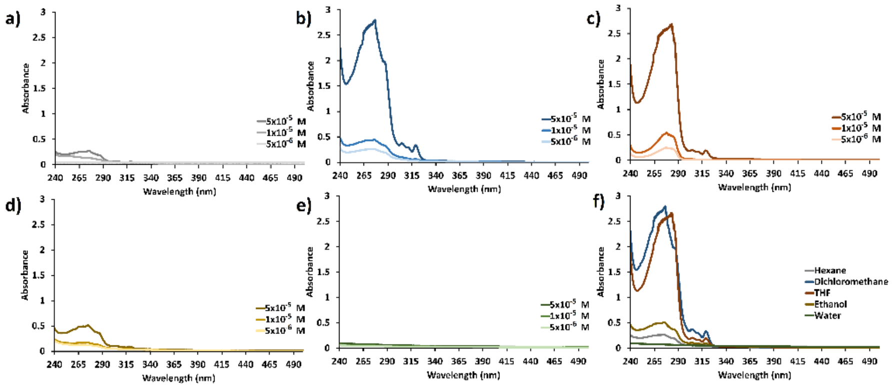

Figure S13. UV-Vis absorption spectra of compound **6** in (a) hexane, (b) DCM, (c) THF, (d) ethanol, and (e) water at various concentrations, and (f) overlap spectra of the solvents at 5×10^{-5} M.

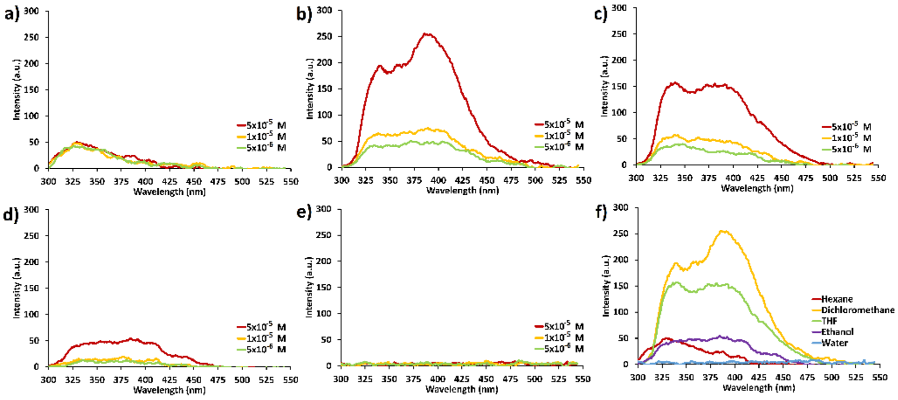

Figure S14. Fluorescence emission spectra of compound **6** in (a) hexane, (b) DCM, (c) THF, (d) ethanol, and (e) water at various concentrations, and (f) overlap spectra of the solvents at 5×10^{-5} M ($\lambda_{exc.}$: 290 nm).

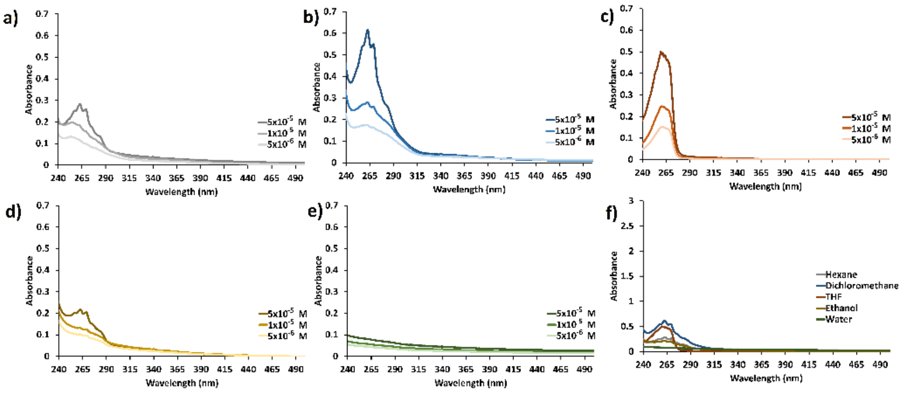

Figure S15. UV-Vis absorption spectra of compound **7** in (a) hexane, (b) DCM, (c) THF, (d) ethanol, and (e) water at various concentrations, and (f) overlap spectra of the solvents at 5 × 10^{-5} M.

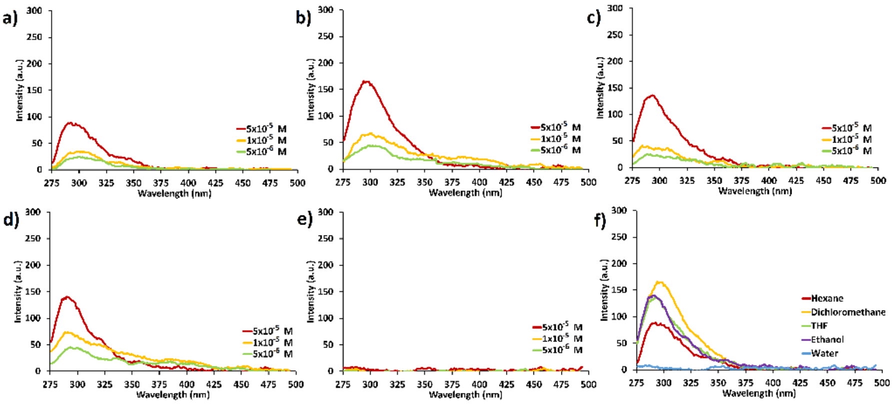

Figure S16. Fluorescence emission spectra of compound **7** in (a) hexane, (b) DCM, (c) THF, (d) ethanol, and (e) water at various concentrations, and (f) overlap spectra of the solvents at 5 × 10^{-5} M ($\lambda_{exc.}$: 260 nm).

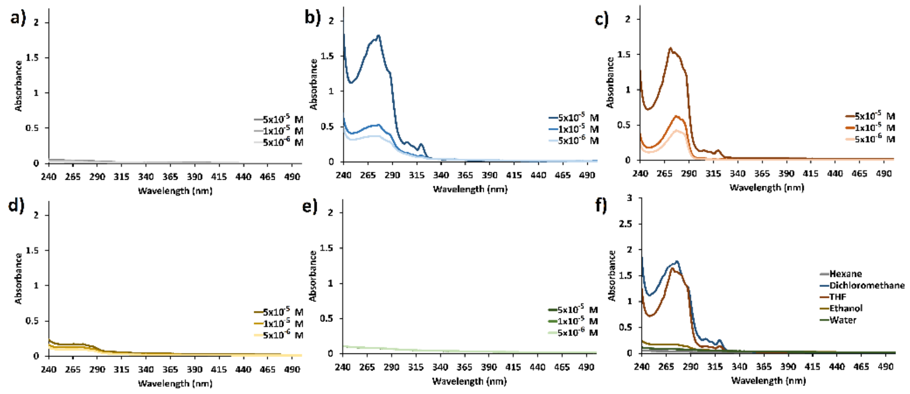

Figure S17. UV-Vis absorption spectra of compound **8** in (a) hexane, (b) DCM, (c) THF, (d) ethanol, and (e) water at various concentrations, and (f) overlap spectra of the solvents at 5 × 10^{-5} M.

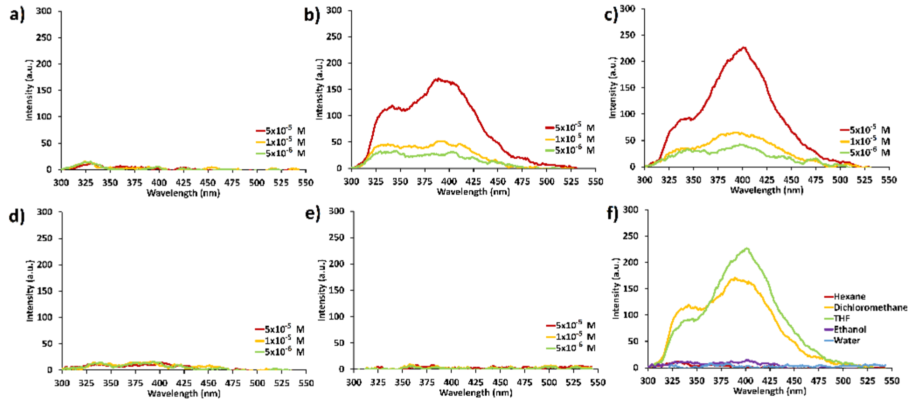

Figure S18. Fluorescence emission spectra of compound **8** in (a) hexane, (b) DCM, (c) THF, (d) ethanol, and (e) water at various concentrations, and (f) overlap spectra of the solvents at 5 × 10^{-5} M ($\lambda_{exc.}$: 290 nm).
